# Costs of management of acute respiratory infections in older adults: A systematic review and meta-analysis

**DOI:** 10.7189/jogh.12.04096

**Published:** 2022-10-30

**Authors:** Shanshan Zhang, Pia Wahi-Singh, Bhanu Wahi-Singh, Alison Chisholm, Polly Keeling, Harish Nair, RESCEU Investigators, Harish Nair, Harry Campbell, Ting Shi, Shanshan Zhang, You Li, Xin Wang, Peter Openshaw, Jadwicha Wedzicha, Philippe Beutels, Louis Bont, Andrew Pollard, Eva Molero, Federico Martinon-Torres, Terho Heikkinen, Adam Meijer, Thea Kølsen Fischer, Maarten van den Berge, Carlo Giaquinto, Rafael Mikolajczyk, Michael Abram, Kena Swanson, Amanda Leach, Sonia Stoszek, Scott Gallichan, Alexia Kieffer, Clarisse Demont, Arnaud Cheret, Sandra Gavart, Jeroen Aerssens, Brian Rosen

**Affiliations:** 1 Centre for Global Health, Usher Institute, University of Edinburgh, Edinburgh, UK; 2 Department of Preventive Dentistry, Peking University, School and Hospital of Stomatology, Beijing, China; 3 ReSViNET Foundation, Zeist, theNetherlands; 4 MRC/Wits Rural Public Health and Health Transitions Research Unit (Agincourt), School of Public Health, Faculty of Health Sciences, University of Witwatersrand, Johannesburg, South Africa

## Abstract

**Background:**

Acute respiratory infections (ARIs) accounted for an estimated 3.9 million deaths worldwide in 2015, of which 56% occurred in adults aged 60 years or older. We aimed to identify the cost of ARI management in older adults (≥50 years) in order to develop an evidence base to assist decision-making for resource allocation and inform clinical practice.

**Methods:**

We searched 8 electronic databases including Global Health, Medline and EMBASE for studies published between January 1, 2000 and December 31, 2021. Total management costs per patient per ARI episode were extracted and meta-analysis was conducted by World Health Organization (WHO) region and World Bank income level. All costs were converted and inflated to Euros (€) (2021 average exchange rate). The quality of included studies and the potential risk of bias were evaluated.

**Results:**

A total of 42 publications were identified for inclusion, reporting cost data for 8 082 752 ARI episodes in older adults across 20 countries from 2001 to 2021. The majority (86%) of studies involved high-income countries based in Europe, North America and Western Pacific. The mean cost per episode was €17 803.9 for inpatient management and €128.9 for outpatient management. Compared with costs reported for patients aged <65 years, inpatient costs were €154.1, €7 018.8 and €8 295.6 higher for patients aged 65-74 years, 75-84 years and over 85 years. ARI management of at-risk patients with comorbid conditions and patients requiring higher level of care, incurred substantially higher costs for hospitalization: €735.9 and €1317.3 respectively.

**Conclusions:**

ARIs impose a substantial economic burden on health systems, governments, patients and societies. This study identified high ARI management costs in older adults, reinforcing calls for investment by global health players to quantify and address the scale of the challenge. There are large gaps in data availability from low-income countries, especially from South East Asia and Africa regions.

Acute respiratory infections (ARIs) accounted for an estimated 3.9 million deaths worldwide in 2015, of which 56% were in adults aged 60 years or older [1]. The global reduction in paediatric lower respiratory infection mortality illustrates the potential effectiveness of public health interventions such as increased vaccine coverage for Streptococcus pneumoniae and *Haemophilus influenzae* type B. To reduce the age-related inequalities in public health priorities, attention and resources must now extend to the persistent and growing ARI-related burden in the world’s ageing and increasingly older populations. We have previously published a systematic review and meta-analysis on cost of management of severe pneumonia in young children; however, there are no similar reviews in adults [2].

The infectious acute respiratory disease burden needs to be estimated to further inform use of vaccines to improve healthy years in older populations [3,4]. It is well acknowledged that older adults (≥50 years) are more susceptible to infections in part due to immunosenescence. There is also an associated risk of high use (and potential misuse) of antibiotics in older adults that warrants attention as part of global stewardship efforts to address the challenges posed by antimicrobial resistance [5]. The need to qualify and quantify the cost of infectious disease burden in older adults was outlined as a necessary activity. Robust evidence of the health, societal and economic burden posed by ARIs in older and at-risk populations are required to prioritize future research investment with respect to current and emerging vaccines and therapeutics. Greater understanding of the costs associated with the management of ARIs is required to guide public health agencies, industry, national and global health policy makers for appropriate resource allocation and priority setting.

Therefore, we conducted a systematic review and meta-analysis of published literature with the aim of identifying the cost (per episode) of management of all-cause ARI in older adults by regions, classified by type of comorbidity and/or intervention for the period prior to COVID-19 pandemic.

## METHODS

### Search strategy

A review protocol was developed but not registered on publicly available database. We reported our findings in line with the recommendations of Preferred Reporting Items for Systematic reviews and Meta-Analyses (PRISMA) guidelines and incorporated expert recommendations for conducting high-quality systematic reviews of economic evaluations [6]. Searches were conducted in 8 global health databases in order to achieve maximum coverage of the published literature: MEDLINE, Embase, NHS EED, EconLit (EBSCO) and Web of Science bibliometric databases, CAB Abstracts, Global Health, Global Index Medicus, with the grey literature guided by the subject of the review and manually searched reference lists of eligible articles. The eight databases were used to ensure inclusion of a wide range of health literature as well as publications relevant to global health from less mainstream sources, and relevant economic records not otherwise published in biomedical science journals. We included studies published in English, Spanish, French and Chinese languages, to achieve maximum coverage of related articles.

### Inclusion and exclusion criteria

General search headings applied were: “Acute Respiratory Infections”, “ARIs”, and “Economics” (detailed search strategy for individual databases are available in Table S1 in the **Online Supplementary Document**). We included eligible publications reporting empirical cost data of ARI management in older adults (≥50 years) published between January 1, 2000 and December 31, 2021 (capturing 20 years’ worth of data as is common is burden of disease studies). Two of the contributing authors (PWS & BWS) independently screened and evaluated the titles and abstracts of all records retrieved and checked the reference lists of eligible articles for further studies. Any disagreements were arbitrated by the lead author (SZ). The inclusion and exclusion criteria are shown in Table S2 in the **Online Supplementary Document**.

### Data extraction

We extracted data on cost per patient, per ARI episode and the overall cost of ARI inpatient and outpatient management. Cost per episode included direct medical, non-medical and indirect costs of ARI management. Direct medical costs included costs related to medication, diagnostic tests, medical staff time and hospital stay. Direct non-medical costs included those related to food, transportation and accommodation. Any additional data on indirect costs such as caregivers’ time and productivity losses were also recorded, where available. Five researchers (SZ, AC, PK, PWS, BWS) extracted these data independently, and final data extraction was crosschecked by the lead author (SZ). The extracted raw data are available on Edinburgh DataShare (https://datashare.ed.ac.uk/handle/10283/4482).

Intervention costs were first converted to local currency, when needed, for the stated price year of study, and inflated to its 2021 original currency value using the gross domestic product (GDP) deflator index in respective years from the International Monetary Fund (IMF) World Economic Outlook database [7]. All costs were then converted to their equivalent price in 2021 Euro (€) based on the purchasing power parity of GDP (period average in 2021) for the stated price year of the study [8].

### Statistical analysis

Costs were reported as global averages and stratified by World Health Organization (WHO) regions and World Bank (WB) income regions. Cost per episode, cost by component (direct medical, direct non-medical and indirect costs) and percentage of total costs per episode in each component were summarized. Relative estimates for at-risk subgroups were extracted from each study. Based on data generated for each study, meta-estimates of cost per episode and weighted mean differences between groups were quantified using a sample size weighted, random effect model of meta-analysis (metan command) in Stata Version 17.0 (StataCorp, College Station, Texas, USA). For each subgroup of ARI management, we summarized the data and reported a point estimate and 95% confidence interval (CI) or cost range for the cost per hospitalization, where appropriate. Statistical heterogeneity was evaluated using *I^2^* statistic. Publication bias was assessed using Egger’s test.

### Quality assessment

The quality of eligible records was evaluated using a 13-item modified Drummond checklist that focused on aspects of methodological robustness and reporting detail [9-12]. The potential risk of bias was assessed using The Consensus Health Economic Criteria (CHEC) list [9-11]. (Table S3 and Table S4 in the **Online Supplementary Document**).

## RESULTS

### Search results

**Figure 1:**
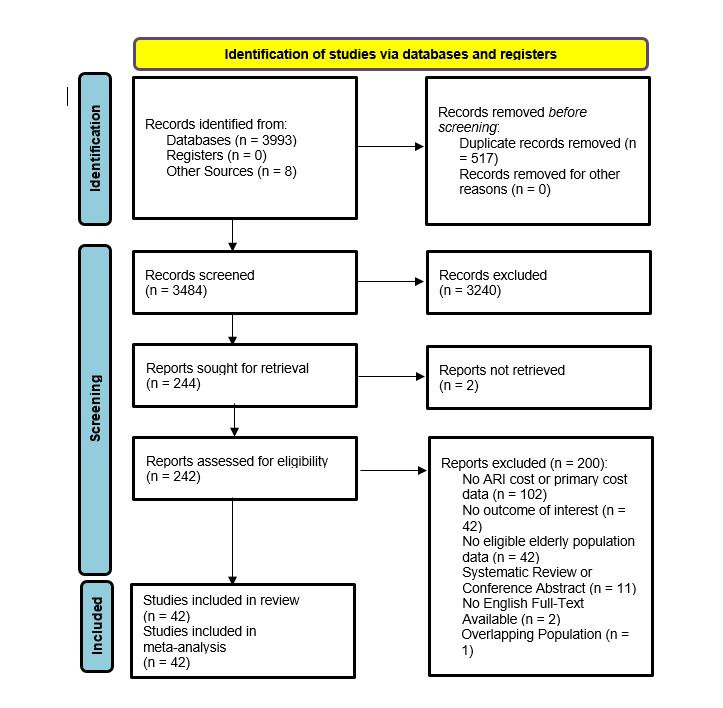
Search results Preferred Reporting Items for Systematic reviews and Meta-Analyses (PRISMA) flowchart. ARI – acute respiratory infection.

The initial search strategy identified 4001 records from 8 databases (3484 excluding duplicates). Following manual screening of relevant titles, abstracts and full text articles (**Figure 1**), a total of 42 published studies was considered eligible and included in the final systematic review [13-54].

### Study characteristics

A total of 8 082 752 disease episodes were included in the cost analysis, including 7 257 134 inpatient cases and 825 618 outpatient and emergency cases. The mean sample size of included studies was 192 446 (Range 60-12,447,087). Study characteristics are summarized in Table S5 in the **Online Supplementary Document**.

A total of 42 published peer-reviewed articles were included in the final review. The majority of the eligible papers (n = 33, 78.6%) reported empirical costs for pneumonia, 9.5% (n = 4) reported costs related to ARIs in general and 7.1% (n = 3) reported costs for influenza-like illness (ILI) and 4.8% (n = 2) for respiratory syncytial virus (RSV) (**Table 1**).

**Table 1:** Study characteristics summary

Study characteristics	Categories	n (% of total)
**WHO region**	European region	15 (35.71%)
	The Americas region	16 (38.10%)
	West Pacific region	10 (23.81%)
	South East Asia region	1 (2.38%)
**World Bank income level**	High-income level	36 (85.71%)
	Upper-middle-income level	4 (9.52%)
	Lower-middle-income level	2 (4.76%)
**Type of study**	Cost of illness	39 (92.86%)
	Cost-effectiveness analysis	2 (4.76%)
	Cost-minimization analysis	1 (2.38%)
**Study design**	Prospective cohort study	9 (21.43%)
	Retrospective cohort study	31 (73.81%)
	Case-control study	1 (2.38%)
	Cross-sectional study	1 (2.38%)
**Perspective**	Healthcare (unspecified)	25 (59.52%)
	Healthcare (payer/provider/employer)	10 (23.81%)
	Societal	6 (14.29%)
	Both societal and health care perspectives	1 (2.38%)
**Conditions**	Pneumonia	33 (78.57%)
	Acute respiratory infection	4 (9.52%)
	Influenza-like illness	3 (7.14%)
	Respiratory syncytial virus	2 (4.76%)
**Gender distribution**	Reported*	32 (76.19%)
	Not reported	10 (23.81%)
**Comorbidities**	Reported	28 (66.67%)
	Not reported	14 (33.33%)

*Percentage male range 20%-76% across included publications.

The 42 eligible papers contained empirical cost data (related to ARI management) from 20 countries. Stratification of publication coverage by WHO and WB income regions revealed that the majority of the economic evaluations took place in high-income countries (HICs, n = 36, 85.7%) and from either Europe (35.7%) or North America (38.1%). USA contributed the maximum number of papers for any individual country (n = 15, 35.7%). Of the 6 WHO regions, we were unable to obtain any data from the African and Eastern Mediterranean regions. Despite accounting for more than three-quarters (81%) of the World’s population, South America, Africa, Eastern Mediterranean, South East Asia and Western Pacific region countries were un- or under-represented in the literature, accounting for only 26.2% of eligible papers, collectively (**Table 2**).

**Table 2:** Global coverage of the included studies

WHO region (number of studies, % of total)	Income level (number of studies, % of total)	Countries (number of Studies, % of total)
European Region (15, 35.71%)	High-income (12, 28.57%)	France (3, 7.14%)
		Germany (1, 2.38%)
		Italy (1, 2.38%)
		Netherlands (2, 4.76%)
		Spain (2, 4.76%)
		United Kingdom (2, 4.76%)
		Czech Republic, Slovakia, Poland, and Hungary (1, 2.38%)
	Upper-middle income (3, 7.14%)	Turkey (3, 7.14%)
The Americas Region (16, 38.10%)	High-income (16, 38.10%)	Canada (1, 2.38%)
		United States of America (15, 35.71%)
West Pacific Region (10, 23.81%)	High-income (8, 19.05%)	Hong Kong (1, 2.38%)
		Japan (3, 7.14%)
		New Zealand (1, 2.38%)
		Republic of Korea (3, 7.14%)
	Upper-middle-income (1, 2.38%)	China (1, 2.38%)
	Lower-middle-income (1, 2.38%)	Vietnam (1, 2.38%)
South East Asia Region (1, 2.38%)	Lower-middle-income (1, 2.38%)	India (1, 2.38%)

All eligible studies included populations with an average age of ≥50 years (including mean age minus SD over 50 years) or reported age-stratified costs and included at least one subgroup aged ≥50 years. The mean age of the included population ranged from 48.0 to 87.8 years, with 67% (n = 28) of studies including a population of mean age over 65 years. Gender distribution was reported in most studies (n = 32, 76.2%), with men accounting for 20 to 76% in different study populations. Two-thirds of the papers (n = 28, 66.7%) reported some level of comorbidity data, most commonly related to prevalence of chronic comorbid conditions, notably: chronic obstructive pulmonary disease (COPD, 4.1%-64.0%) and asthma (5.0%-20.4%), diabetes mellitus (11.0%-50.0%) and cardiovascular disease (CVD, 6.1%-36.0%).

Cost-of-illness (COI) studies accounted for 92.9% (n = 39) of the eligible papers; cost-effectiveness and cost-minimization for the remaining 4.7% and 2.4%, respectively. The majority of studies (n = 25, 59.5%) considered management costs from a health care system perspective, but the payer perspective was incorporated in 23.8% of papers (n = 10) and the societal perspective in 14.3% of papers (n = 6). Electronic health record and insurance databases were the most common sources of cost data.

Of a maximum score of 13 using the modified Drummond tool, the average quality score was 7.1 points (standard deviation 1.3, range 5-9) [12]. The potential for bias of included studies was generally medium (n = 20, 47.6%) or high (n = 21, 50.0%) (Table S6 and Table S7 in the **Online Supplementary Document**). Quality domains that contributed to the downgrading of papers included: lack of separate reporting of resource quantities and unit costs; limited description of data sources and reporting of only inferential information about the time horizon used. Some quality items lacked direct relevance to descriptive COI studies, among them: comparative outcomes for two alternatives and clear description of statistical tests. There was no publication bias (*P* = 0.082).

### Cost per patient for ARI management

The global overall weighted mean cost per inpatient episode was €17 803.9 (range =  €154.6- €30 068.5) (**Table 3**). Regional cost data showed that inpatient cost was €1187.6 per episode in the European Region. Inpatient cost in the Region of Americas (AMR) was €17 833.6, the highest, followed by the Western Pacific Region (WPR), which was €2455.2 per episode. ARI management costs were also higher in high income countries (€17 805.5) compared to middle income countries (€1275.0 in upper middle income countries and €221.2 in lower middle income countries). There were no cost data available from low income countries.

**Table 3:** Weighted mean cost by WHO region and income level

Weighted mean cost per episode by WHO region and income level	Number of ARI, episode (% of total in each category)	Weighted mean cost	(Cost range)
**Inpatient EUR**	317 983 (4.4%)	1187.6	(546.3-21 471.4)
AMR	6 894 046 (95.0%)	17 833.6	(1022.0-30 068.5)
WPR	44 945 (0.06%)	2455.2	(323.1-8271.1)
SEAR	160 (0.04%)	166.0	(154.6-368.0)
High income countries	7 255 798 (99.9%)	17 805.5	(546.3-30 068.5)
Upper middle income countries	1018 (0.01%)	1275.0	(323.1-2340.1)
Lower middle income countries	318 (0.004%)	221.2	(154.6-452.1)
Overall	7 257 134 (100.0%)	17 803.9	(154.6-30 068.5)
**Outpatient EUR**	645 362 (78.2%)	131.1	(53.9-968.1)
AMR	165 675 (20.1%)	492.3	(359.6-2493.9)
WPR	14 450 (1.7%)	229.7	(229.7-229.7)
SEAR	131 (0.02%)	5.6	(5.6-10.2)
High income countries	825 336 (99.97%)	141.6	(53.9-2493.9)
Upper middle income countries	151 (0.02%)	140.5	(140.5-140.5)
Lower middle income countries	131 (0.02%)	5.6	(5.6-10.2)
Overall	825 618 (100.0%)	128.9	(5.6-2493.9)

EUR – European Region, AMR – Region of The Americas, WPR – Western Pacific Region, SEAR – South East Asia Region, EMR – Eastern Mediterranean Region

For outpatient management cost, the global overall weighted mean was €128.9 (range =  €5.6, €2493.9). A similar trend was observed in outpatient costs to inpatient costs, with the highest outpatient cost being in AMR (€492.3) followed by WPR (€229.7). Outpatient management mean cost in high income countries and middle income countries was €141.6 and €140.5, respectively.

Direct and indirect non-medical costs were reported in 2 studies. Direct non-medical costs (mainly food and transportation) were reported to contribute 0.1% of the total management cost per patient and indirect costs, representing loss of productivity, were reported to contribute 29.4%-40.7% of the total management cost.

Subgroup costs differences were calculated by weighted mean difference (**Table 4**). The cost for patients aged over 65 years was €154.1 (95%CI = €100.7-€157.5) higher compared to patients under 65 years. The older age group from 75-84 years cost €7018.8 more, but for the patient group aged over 85 years, the cost difference was larger at €8295.6. This trend has been observed in many studies.

**Table 4:** Inpatient cost differences between different groups

Subgroup	Weighted mean cost difference (2021 €)	95% CI
**Age band**		
66-74y vs ≤65y	154.1	(100.7-157.5)
75-84y vs ≤65y	7018.8	(6994.3-7043.3)
>85y vs ≤65y	8295.6	(8284.9-8306.4)
**Initial admission vs readmission**	1649.4	(1203.5-2095.2)
**ICU vs general wards**	9857.0	(9768.8-9945.2)
Risk level*		
Moderate risk vs low risk	735.9	(645.4-826.5)
High risk vs low risk	1317.3	(1121.4-1513.3)
**Death in hospital vs successfully treated**	1150.8	(1100.5-1201.1)

y – years, ICU – intensive care unit

*Risk level – moderate-risk: patients with one or more chronic conditions, eg, congestive heart failure, coronary artery disease, diabetes, COPD, asthma, liver disease; high-risk: patients with immuno-compromising conditions, eg, HIV, neoplasm, chronic renal failure, organ transplant; low-risk: patients who did not belong in moderate- or high-risk categories.

ARI management costs rise as the level of risk and level of care increases. Patients with one or more chronic conditions, eg, congestive heart failure, coronary artery disease, diabetes, COPD, asthma, liver disease, were assigned to the moderate-risk group, patients who were immunocompromised (eg, HIV, neoplasm, chronic renal failure, organ transplant) were assigned to the high-risk category, and patients who did not belong in the moderate- or high-risk categories were assigned to the low-risk category. Management costs for moderate-risk patients were €735.9 higher than low-risk patients, and the high-risk group cost €1317.3 more than the low-risk group. ICU care was €9857.0 (95 CI% = 9768.8-9945.2) more expensive than non-ICU care. The initial hospital admission costs were €1649.4 (95% CI = 1203.5-2095.2) higher than hospital readmission.

## DISCUSSION

### Principal findings

This is the first systematic review and meta-analysis of the cost of ARI management in older adults which summarizes the empirical cost management estimates for an ARI episode in adults aged 50 years and over in 20 countries worldwide. It demonstrates that the economic burden associated with ARI in older adults is substantial. Community-acquired pneumonia (CAP) COI studies made up the majority of the ARI literature. More than three-quarters of studies were conducted in HICs within Europe and North America where inpatient costs were nearly 14 times higher than in middle-income countries. Inpatient costs varied substantially between and within global regions, ranging from €166.0 in South-East Asia to €1187.6 in Europe and €17 833.6 in North America.

A trend of cost increase was observed in elderly patients aged over 65 years, with those aged 75 to 84 years generating more costly hospitalization episodes than younger groups: weighted mean difference of €7018.8 (95% CI =  €6994.3-€7043.3) for the 75-84 group compared with the under 65 group. This may be because patients in the older age band had a higher prevalence of underlying diseases and more severe infections and may have required longer hospital stays and ICU admission. As reported in this study, patients with comorbidities were €1317.3 more costly than the healthy adults and costs related to ICU admission were €9857.0 higher compared to admission in general inpatient wards. A patient who died in the hospital generated €1150.8 more cost than those successfully treated in the hospital. The estimated costs reported here are likely substantial under-estimates as it is likely that a proportion of ARIs may not be accounted for even in high income settings. Beyond the direct and indirect costs associated with management of the ARI episode, there are larger economic implications at societal level – patients in this age group may not fully recover following the ARI episode and experience a decline in functional status following ARI and thus require additional care to conduct daily assistance [55]; there is economic impact beyond loss in productivity from those still in workforce; and widespread use of antibiotics for ARI that are primarily of viral aetiology has an impact on antimicrobial resistance.

As older adults within the 50-to-64-year age range are still of working age, the indirect costs incurred by loss of productivity due to ARI account for a large proportion (around 40%) of the total episode costs. This may, in turn, result in a disproportionate burden (costs) to family and society. However, these costs were mostly reported from high-income countries. Data for low-income countries were missing but coping with considerable costs of ARI management in middle- and low- income countries can lead to catastrophic consequences and impoverishment for the affected families [56]. More cost studies are needed to better understand the impact and the possible benefits of adequate prevention.

To help optimize the cost-effectiveness of future vaccination programmes and associated interventions, economic evaluations of ARIs would benefit from reporting of pathogen-specific management costs. In the short-term, this could be achieved by prospective pathogen testing and cost collection in small (eg, single centre) studies, which could provide weighting factors (of average costs) for relative pathogen-specific cost impact modelling. In addition, embedding COI variables within the WHO’s RSV and influenza surveillance programmes [57] could provide valuable evidence to address this limitation of the current ARI cost literature [58].

The inclusive design of this review, which included retrospective and prospective cohort and observational studies as well as randomized controlled trials (RCTs), ensured identification of empirical costs incurred within routine ecologies of care and everyday patient populations. This highlights the wide scope of allied costs related to ARI (at regional and country levels) due to differences in management styles and cost reporting methods around the world. Regardless of the setting and methodological heterogeneity, our data underline the urgent need for effective means of ARI prevention strategies and timely intervention.

There are a number of limitations in the review methodology that should be acknowledged to inform future research efforts and aid in the appropriate interpretation of the data for further use. First, less than half of included publications specified the exact range of costs including mean and median costs. Adequate representation of cost sample data should always include SD or 95% of reported mean or interquartile range of median. Second, the methods for case ascertainment differed significantly from study to study; they were mostly ICD-9 and ICD-10 codes in health records, but this variability may result in underreporting. Third, the variation in management costs reported in the literature reflects not only differences in economies, but also in health systems, patient populations (eg, case definitions) and study design. Care must be taken when interpreting the data and considering its use within cost modelling. The baseline comorbidity status (eg, COPD) should be captured as the cost for high-risk groups would potentially increase. The differences in costs reported from different sources and the methodological standardization achieved in this current study provide a useful roadmap for future research, which is urgently needed especially for low-income countries and underrepresented regions (ie, South East Asia, Africa and South America). Fourthly, we identified and included two studies from LMIC region (ie, South East Asia). Therefore, our estimates for LMICs are likely uncertain and biased. Fifth, we have not analysed for risk of bias by the funding source for each of the included studies. Finally, this article covers the cost data before COVID-19 pandemic, which may underestimate the cost for ARI in elderly population during the inter and post pandemic period.

Comparing costs between different countries is difficult as health care systems, resource prices, diagnostics, treatment standards and incentives vary [59]. Nevertheless, these data have several important implications for public health policy, practice and research. From a public health policy perspective, this review emphasises the importance of robust primary and secondary prevention in older adults, to reduce hospital length of stay (LOS) and related management costs [60]. Primary prevention efforts should include immunization programmes (and coverage) where/when vaccines are available [61].

Depending on where the infection is treated, provision of services to support management within the community and outpatient settings has the potential for substantial cost savings: hospital-based community-acquired pneumonia management is estimated to be 8 times as costly as outpatient care and 12 times as costly as primary care management [13,32]. When admission is required, strategies to avoid further complications and use of intensive care services should be implemented to reduce the impact on patients’ quality of life as well as their health care resource utilisation and associated costs [14,19,33]. In this study, different cost categories for at risk groups were substantially confounded by indication. Strategies to ensure use of evidence-based interventions that reduce length of stay and complication risk and tailoring therapy to the individual patient or implicated pathogen, also hold the potential for further cost savings [34]. An ageing population globally (and not just in HICs) and the high costs associated with ARI management costs in older adults reinforces the need for investment by all global health players to quantify and address the scale of the challenge.

## CONCLUSIONS

This study identified high ARI management costs in older populations, reinforcing calls for investment by all global health players to quantify and address the scale of the challenge. Of relevance for future research and planning, there are large gaps in data availability from low-income countries, especially from South East Asia and African Region.

## Additional material


Online Supplementary Document
