# Supplementary material for: Costs of management of acute respiratory infections in older adults: A systematic review and meta-analysis
Source: J Glob Health. 2022 Oct 30;12:04096. doi: 10.7189/jogh.12.04096 (PMC9639666; doi:10.7189/jogh.12.04096)
Supplement: Online Supplementary Document [file jogh-12-04096-s001.pdf]

Online Supplementary Document

Title: Costs of management of acute respiratory infections in older adults: a systematic review and meta-analysis

Shanshan Zhang [1,2], Pia Wahi-Singh[1], Bhanu Wahi-Singh[1]

Alison Chisholm[1], Polly Keeling[1], Harish Nair [†,1,3], for the RESCEU Investigators.

† Corresponding author

1. Centre for Global Health, Usher Institute, University of Edinburgh, Edinburgh, UK
2. Department of Preventive Dentistry, Peking University, School and Hospital of Stomatology, Beijing, China
3. ReSViNET Foundation, Zeist, The Netherlands

†Correspondence: Medical School, Teviot Place, Edinburgh EH8 9AG, United Kingdom (Harish.Nair@ed.ac.uk).

RESCEU Investigators

Harish Nair, Harry Campbell, Ting Shi, Shanshan Zhang, You Li, Xin Wang (University of Edinburgh); Peter Openshaw, Jadwicha Wedzicha (Imperial College London); Philippe Beutels (Universiteit Antwerpen); Louis Bont (University Medical Centre Utrecht); Andrew Pollard (University of Oxford); Eva Molero (Team-IT); Federico Martinon-Torres (Servicio Galego de Saude); Terho Heikkinen (Turku University Central Hospital); Adam Meijer (National Institute for Public Health and the Environment); Thea Kølsen Fischer (Statens Serum Institut); Maarten van den Berge (Academisch Ziekenhuis Groningen); Carlo Giaquinto (Fondazione PENTA for the treatment and care of children with HIV-ONLUS); Rafael Mikolajczyk (Martin-Luther University Halle-Wittenberg); Michael Abram (AstraZeneca); Kena Swanson (Pfizer); Amanda Leach, Sonia Stoszek (GlaxoSmithKline); Scott Gallichan, Alexia Kieffer, Clarisse Demont (Sanofi Pasteur); Arnaud Cheret, Sandra Gavart, Jeroen Aerssens, (Janssen); Brian Rosen (Novavax).

Table S1. Search strategy

Table S1-a MEDLINE (Accessed from: Ovid MEDLINE(R) 1946 to February Week 3 2022)

#	Search Term (s)
1	Respiratory Syncytial Virus Infections/
2	respiratory syncytial viruses.tw,ab.
3	respiratory syncytial virus, human.tw,ab.
4	(respiratory syncytial vir* or rsv).tw.
5	Respiratory Tract Infections/
6	(acute respiratory infection* or acute respiratory tract infection*).tw.
7	("lower respiratory tract infection*" or lrti* or tract "upper respiratory infection*" or urti*).tw,ab.
8	exp Bronchiolitis/
9	bronchiolit*.tw.
10	pneumonia/
11	pneumonia, viral/
12	pneumon*.tw.
13	1 or 2 or 3 or 4 or 5 or 6 or 7 or 8 or 9 or 10 or 11 or 12
14	exp Asthma/
15	(asthma$ or wheez$).ti,ab.
16	(asthmatic? or (asthma$ adj2 (chronic$ or patient?))).ab.
17	Bronchial Hyperreactivity/
18	bronchial$ hyperreactivit$.ti,ab.
19	lung diseases, obstructive/
20	(obstructive adj (lung disease or lung diseases)).ti.
21	14 or 15 or 16 or 17 or 18 or 19 or 20
22	Lung Diseases, Obstructive/
23	exp Pulmonary Disease, Chronic Obstructive/

24	emphysema$.mp.
25	(chronic$ adj3 bronchiti$).mp.
26	(obstruct$ adj3 (pulmonary or lung$ or airway$ or airflow$ or bronch$ or respirat$)).mp.
27	COPD.mp.
28	COAD.mp.
29	COBD.mp.
30	(exacerbation* or exacerb* or attack or "acute worsen*").mp,tw,ab,ti.
31	22 or 23 or 24 or 25 or 26 or 27 or 28 or 29
32	(21 or 31) and 30
33	13 or 32
34	exp "Costs and Cost Analysis"/
35	Economics, Nursing/
36	Economics, Medical/
37	Economics, Pharmaceutical/
38	exp Economics, Hospital/
39	exp "Fees and Charges"/
40	exp Budgets/
41	budget*.ti,ab,kf.
42	(economic* or cost or costs or costly or costing or price or prices or pricing or pharmacoeconomic* or pharmaco-economic* or expenditure or expenditures or expense or expenses or financial or finance or finances or financed).ti,kf.
43	(economic* or cost or costs or costly or costing or price or prices or pricing or pharmacoeconomic* or pharmaco-economic* or expenditure or expenditures or expense or expenses or financial or finance or finances or financed).ab.

44	(cost* adj2 (effective* or utilit* or benefit* or minimi* or analy* or outcome or outcomes)).ab,kf.
45	(value adj2 (money or monetary)).ti,ab,kf.
46	34 or 35 or 36 or 37 or 38 or 39 or 40 or 41 or 42 or 43 or 44 or 45
47	33 and 46
48	adult/
49	exp aged/
50	middle aged/
51	young adult/
52	adult?.ti,ab,hw.
53	middle aged.ti,ab.
54	elderly.ti,ab.
55	48 or 49 or 50 or 51 or 52 or 53 or 54
56	47 and 55
57	limit 56 to dt=20001001-20211231 [January 1st, 2000 to Decebmer 31st, 2021]
58	limit 57 to english language
59	limit 58 to (female or humans or male)
**60**	**limit 59 to full text**

Table S1-b EMBASE (Embase 1980 to 2022 Week 08)

#	Search Term (s)
1	respiratory syncytial virus infection.de.
2	respiratory syncytial pneumovirus.de.
3	respiratory syncytial virus.ab,ti.
4	respiratory syncytial viruses.ab,ti.
5	rsv.ab,ti.
6	respiratory tract infection.de.
7	acute respiratory infection.ab,ti.
8	acute respiratory infections.ab,ti.
9	lower respiratory tract infection.ab,ti.
10	upper respiratory tract infection.ab,ti.
11	lower respiratory tract infections.ab,ti.
12	upper respiratory tract infections.ab,ti.
13	ari.ab,ti.
14	lrti.ab,ti.
15	urti.ab,ti.
16	pneumonia.de.
17	virus pneumonia.de.
18	pneumon*.ab,ti.
19	1 or 2 or 3 or 4 or 5 or 6 or 7 or 8 or 9 or 10 or 11 or 12 or 13 or 14 or 15 or 16 or 17 or 18
20	exp *asthma/
21	(asthma$ or wheez$).ti.
22	(asthma$ adj3 (sever$ or chronic$ or primary or major)).ab.
23	80 or 81 or 82

24	exp *Pulmonary Disease, Chronic Obstructive/
25	Lung Diseases, Obstructive/
26	(chronic$ adj3 bronchiti$).ab.
27	(obstruct$ adj3 (pulmonary or lung$ or airway$ or airflow$ or bronch$ or respirat$)).ab.

28	emphysema$.ab.
29	COPD.mp.
30	COAD.mp.
31	COBD.mp.
32	(exacerbation* or exacerb* or attack* or "acute worsen*").mp.
33	24 or 25 or 26 or 27 or 28 or 29 or 30 or 31
34	(23 or 33) and 32
35	19 or 34
36	Economics/
37	Cost/
38	exp Health Economics/
39	Budget/
40	budget*.ti,ab,kw.
41	(economic* or cost or costs or costly or costing or price or prices or pricing or pharmacoeconomic* or pharmaco-economic* or expenditure or expenditures or expense or expenses or financial or finance or finances or financed).ti,kw.
42	(economic* or cost or costs or costly or costing or price or prices or pricing or pharmacoeconomic* or pharmaco-economic* or expenditure or expenditures or expense or expenses or financial or finance or finances or financed).ab. /freq=2
43	(cost* adj2 (effective* or utilit* or benefit* or minimi* or analy* or outcome or outcomes)).ab,kw.

44	(value adj2 (money or monetary)).ti,ab,kw.
45	36 or 37 or 38 or 39 or 40 or 41 or 42 or 43 or 44
46	45 and 45
47	adult?.ti,hw.
48	Elderly?.ti,hw.
49	47 or 48
50	46 and 49
51	limit 50 to dc=20000101-20211231
52	limit 51 to (human and male and female)
53	limit 52 to english language
**54**	**limit 53 to full text**

Table S1-c

Web Of Science (https://www-webofscience-com.ezproxy.is.ed.ac.uk/wos/woscc/basic-search)

#	Search Term (s)	Search Options

1	(TS=("respiratory syncytial virus infection") OR TS=("respiratory syncytial pneumovirus"/) OR TS=("respiratory syncytial virus":) OR TI=("respiratory syncytial virus") OR TI=("respiratory syncytial viruses") OR TS=(rsv) OR TI=(rsv) OR TS=("acute respiratory infection") OR TS=("acute respiratory infections") OR TS=("lower respiratory tract infection") OR TS=("upper respiratory tract infection") OR TI=("acute respiratory infection") OR TI=("acute respiratory infections") OR TI=("lower respiratory tract infection") OR TI=("upper respiratory tract infection") OR TS=(ARI OR LRI OR URI OR ALRTI OR LRTI OR URTI) OR TI=(ARI OR LRI OR URI OR ALRTI OR LRTI OR URTI))	LANGUAGE: (English) Indexes=SCI-EXPANDED, SSCI, A&HCI, CPCI-S, CPCI-SSH, BKCI-S, BKCI-SSH, ESCI, CCR-EXPANDED, IC Timespan= 2000-01-01 to 2021-12-31
2	((((((((((TS=(asthma)) OR (TS=(asthma*))) OR (TS=(wheez*))) OR (TS=((asthma*) ND ((((sever*) OR (chronic*)) OR (primary)) OR (major))))) OR (TS=("pulmonary disease"))) OR (TS=("chronic obstructive pulmonary disease '))) OR (TS=("obstructive lung disease*"))) OR (TS=((chronic*) (bronchiti*)))) OR (TS=((obstruct*) AND ((((((pulmonary) OR (lung*)) OR (airway*)) OR (airflow*)) OR (bronch*)) OR (respiratory*))))) OR (TS=(emphysema*))) OR (TS=((((COPD) OR (coad)) OR (cobd)) OR (aecb))))	LANGUAGE: (English) Indexes=SCI-EXPANDED, SSCI, A&HCI, CPCI-S, CPCI-SSH, BKCI-S, BKCI-SSH, ESCI, CCR-EXPANDED, IC Timespan= 2000-01-01 to 2021-12-31

3	((((((((TS=(economics)) OR (TS=(cost))) OR (TS=((health) (economics)))) OR (TS=(budget))) OR (TI=(budget*))) OR (TS=((((((((((((((((economic*) OR (cost)) OR (costs)) OR (costly)) OR (casting)) OR (price)) OR (prices)) OR (pricing)) OR (pharmacoeconomic*)) OR (pharmaco-economic*)) OR (expenditure)) OR (expenditures)) OR (expense)) OR (expense)) OR (financial)) OR (finance)) OR (finance)) OR (finances)))) OR (TS=((cost*) AND (((((((effective*) OR (utilit*)) OR (benefit*)) OR (minimi*)) OR (analy*)) OR (outcome)) OR (outcomes))))) OR (TS=((value) AND ((money) OR (monetary))))) AND LANGUAGE: (English)	LANGUAGE: (English) Indexes=SCI-EXPANDED, SSCI, A&HCI, CPCI-S, CPCI-SSH, BKCI-S, BKCI-SSH, ESCI, CCR-EXPANDED, IC Timespan= 2000-01-01 to 2021-12-31
4	(TS=(adult* OR Elderly OR aged) NOT TS=(child* OR infant* OR paediat* or pediat* OR baby OR babies OR neonat* OR prenat*)	LANGUAGE: (English) Indexes=SCI-EXPANDED, SSCI, A&HCI, CPCI-S, CPCI-SSH, BKCI-S, BKCI-SSH, ESCI, CCR-EXPANDED, IC Timespan= 2000-01-01 to 2021-12-31
5	(TS=(exacerb* OR attack* OR "acute worsen*"))	LANGUAGE: (English) Indexes=SCI-EXPANDED, SSCI, A&HCI, CPCI-S, CPCI-SSH, BKCI-S, BKCI-SSH, ESCI, CCR-EXPANDED, IC Timespan= 2000-01-01 to 2021-12-31

6	#5 AND #2	Indexes=SCI-EXPANDED, SSCI, A&HCI, CPCI-S, CPCI-SSH, BKCI-S, BKCI-SSH, ESCI, CCR-EXPANDED, IC Timespan= 2000-01-01 to 2021-12-31
7	#6 OR #1	Indexes=SCI-EXPANDED, SSCI, A&HCI, CPCI-S, CPCI-SSH, BKCI-S, BKCI-SSH, ESCI, CCR-EXPANDED, IC Timespan= 2000-01-01 to 2021-12-31
8	#7 AND #3	Indexes=SCI-EXPANDED, SSCI, A&HCI, CPCI-S, CPCI-SSH, BKCI-S, BKCI-SSH, ESCI, CCR-EXPANDED, IC Timespan=2000-01-01 to 2021-12-31
**9**	**#8 AND #4**	**Indexes=SCI-EXPANDED, SSCI, A&HCI, CPCI-S, CPCI-SSH, BKCI-S, BKCI-SSH, ESCI, CCR-EXPANDED, IC Timespan=**2000-01-01 to 2021-12-31

Table S1-d CAB Abstracts and Global Health (Global Health)

#	Search Term (s)

1	Respiratory Syncytial Virus Infections/
2	respiratory syncytial viruses.tw.
3	respiratory syncytial vir*.tw.
4	respiratory syncytial virus, human.tw.
5	(acute respiratory infection* or acute respiratory tract infection*).tw.
6	(lower respiratory tract infection* or lrti).tw.
7	(upper respiratory tract infection* or urti).tw.
8	exp Bronchiolitis/
9	pneumonia/
10	pneumonia, viral/
11	pneumon*.tw.
12	1 or 2 or 3 or 4 or 5 or 6 or 7 or 8 or 9 or 10 or 11
13	exp Asthma/
14	asthma$.ti. or wheez$.ti,ab.
15	(asthmatic? or (asthma$ adj2 (chronic$ or patient?))).ab.
16	bronchial$ hyperreactivit$.ti,ab.
17	"obstructive lung disease".mp.
18	(obstructive adj (lung disease or lung diseases)).ti.
19	13 or 14 or 15 or 16 or 17 or 18
20	(exacerbation* or exacerb* or attack or "acute worsen*").mp.
21	19 and 20
22	"chronic obstructive pulmonary disease".mp.
23	emphysema$.mp.
24	(chronic$ adj3 bronchiti$).mp.
25	(obstruct$ adj3 (pulmonary or lung$ or airway$ or airflow$ or bronch$ or respirat$)).mp.

26	COPD.mp.
27	COAD.mp.
28	AECOPD.mp.
29	22 or 23 or 24 or 25 or 26 or 27 or 28
30	20 and 29
31	12 or 21 or 30
32	exp food costs/ or exp fixed costs/ or exp unit costs/ or exp health care costs/ or exp estimated costs/ or exp variable costs/ or exp costs/ or exp transport costs/
33	"Costs and Cost Analysis"/
34	Economics, Nursing/
35	Economics, Medical/
36	Economics, Pharmaceutical/
37	Economics, Hospital/
38	"Fees and Charges"/
39	exp Budgets/
40	budget*.ti,ab.
41	(economic* or cost or costs or costly or costing or price or prices or pricing or pharmacoeconomic* or pharmaco-economic* or expenditure or expenditures or expense or expenses or financial or finance or finances or financed).ti.
42	(economic* or cost or costs or costly or costing or price or prices or pricing or pharmacoeconomic* or pharmaco-economic* or expenditure or expenditures or expense or expenses or financial or finance or finances or financed).ab. /freq=2
43	(cost* adj2 (effective* or utilit* or benefit* or minimi* or analy* or outcome or outcomes)).ab.
44	(value adj2 (money or monetary)).ti,ab.
45	32 or 33 or 34 or 35 or 36 or 37 or 38 or 39 or 40 or 41 or 42 or 43 or 44
46	31 and 45

47	adult/
48	exp elderly/
49	middle aged/
50	young adult/
51	adult?.ti,ab,hw.
52	middle aged.ti,ab.
53	elderly.ti,ab.
54	elderly?.ti,ab,hw.
55	47 or 48 or 49 or 50 or 51 or 52 or 53 or 54
56	46 and 55
57	limit 56 to full text & cab abstracts fulltext
58	limit 57 to yr="2000 -2021"
**59**	**limit 58 to english language**

Table S1-e

Global Index Medicus (accessed from: https://www.globalindexmedicus.net/)

#	Search Term (s)
1	((tw:("Respiratory Tract Diseases" OR "Respiratory Tract" OR "acute respiratory" OR "acute respiratory infection" OR "upper respiratory tract" OR "upper respiratory tract infection" OR "lower respiratory tract" OR "lower respiratory tract infection" OR "RSV" OR "respiratory syncytial" OR pneumonia OR lrti OR urti OR ari OR bronchiolitis)) OR (tw:((asthma OR COPD OR "chronic obstructive" OR "pulmonary disease" OR "obstructive lung") AND (exacerbation OR exacerb OR attack OR "acute worsening" OR "flare up")))) AND (tw:((Economics OR Cost Or costs OR "cost analysis" OR Fees OR Charges OR Budget OR budgets OR costing OR price OR prices OR pricing OR pharmacoeconomic OR pharmaco-economic OR expenditure OR expenditures OR expense OR expenses OR financial OR finance OR finances OR financed OR "cost-effective" OR "cost effective" OR "cost benefit" or "cost-benefit" OR "cost-utility" or "cost utility" OR value OR money OR monetary)))
2	Limit to Non-MEDLINE<br>[WPRIM (Western Pacific) (1964) ; LILACS (Americas) (1013); IMSEAR (South-EastAsia) (305) ; IMEMR (Eastern Mediterranean) (300) ; AIM (Africa) (53)]
3	Limit to English language records
4	Limit to records relating to adult humans<br>[Humans (331); Adult (86); Aged (84)]
5	Limit to records with full text available
6	Limit to records published between 2000-2021

Econlit (accessed from:

https://web-s-ebscohost-com.ezproxy.is.ed.ac.uk/ehost/search/advanced?vid=0&sid=590fb92ef442-4d63-881c-dd5d234bdfd9%40redis)

#	Search Term (s)	Search Options

Table S1-f

1	AB “respiratory syncytial virus infection” OR AB “respiratory syncytial pneumovirus” OR AB “respiratory syncytial virus” OR AB "respiratory syncytial viruses" OR AB rsv OR AB "acute respiratory tract infection" OR AB "lower respiratory tract infection" OR AB "upper respiratory tract infection" OR AB "acute respiratory infection" OR AB "lower respiratory infection" OR AB "upper respiratory infection" OR AB (ARI OR LRI OR URI OR ALRTI OR LRTI OR URTI)	Limiters - Linked Full Text; Published Date: 20000101-20170631; Search modes - Boolean/Phrase
2	AB asthma OR AB wheeze OR AB "pulmonary disease" OR "chronic obstructive pulmonary disease" OR "obstructive lung disease" OR (chronic AND (obstructive OR pulmonary OR lung OR airway OR airways OR airflow OR respiratory)) OR emphysema OR (COPD OR CORD OR COBD OR AECOPB)	Limiters - Linked Full Text; Published Date: 20000101-20170631; Search modes - Boolean/Phrase
3	AB exacerbation OR exacerb* OR attack	Search modes - Boolean/Phrase; Search modes - Boolean/Phrase
4	S2 AND S3	Search modes - Boolean/Phrase
5	S1 OR S4	Search modes - Boolean/Phrase

NHSEED (accessed from: https://www.crd.york.ac.uk/CRDWeb/)

#	Search Term (s)
1	MeSH DESCRIPTOR Adult EXPLODE ALL TREES
2	MeSH DESCRIPTOR Aged EXPLODE ALL TREES
3	MeSH DESCRIPTOR Child EXPLODE ALL TREES

Table S1-g

4	MeSH DESCRIPTOR Infant EXPLODE ALL TREES
5	MeSH DESCRIPTOR Cost of Illness EXPLODE ALL TREES
6	MeSH DESCRIPTOR Cost of Illness EXPLODE ALL TREES
7	MeSH DESCRIPTOR Cost-Benefit Analysis EXPLODE ALL TREES
8	MeSH DESCRIPTOR Costs and Cost Analysis EXPLODE ALL TREES
9	MeSH DESCRIPTOR Direct Service Costs EXPLODE ALL TREES
10	MeSH DESCRIPTOR Drug Costs EXPLODE ALL TREES
11	MeSH DESCRIPTOR Economics EXPLODE ALL TREES
12	MeSH DESCRIPTOR Employer Health Costs EXPLODE ALL TREES
13	MeSH DESCRIPTOR Health Care Costs EXPLODE ALL TREES
14	MeSH DESCRIPTOR Health Expenditures EXPLODE ALL TREES
15	MeSH DESCRIPTOR Hospital Costs EXPLODE ALL TREES
16	MeSH DESCRIPTOR Technology, High-Cost EXPLODE ALL TREES
17	MeSH DESCRIPTOR Economics, Hospital EXPLODE ALL TREES
18	MeSH DESCRIPTOR Economics, Medical EXPLODE ALL TREES
19	MeSH DESCRIPTOR Economics, Nursing EXPLODE ALL TREES
20	MeSH DESCRIPTOR Economics, Pharmaceutical EXPLODE ALL TREES
21	MeSH DESCRIPTOR Health Care Economics and Organizations EXPLODE ALL TREES
22	MeSH DESCRIPTOR Health Expenditures EXPLODE ALL TREES
23	MeSH DESCRIPTOR Value of Life EXPLODE ALL TREES
24	MeSH DESCRIPTOR Asthma EXPLODE ALL TREES
25	MeSH DESCRIPTOR Pulmonary Disease, Chronic Obstructive EXPLODE ALL TREES
26	(Respiratory Syncytial Virus):TI OR (Respiratory Syncytial Viruses):TI OR (RSV):TI

27	(COPD):TI AND (Exacerbation):TI
28	(asthma):TI AND (Exacerbation):TI
29	(asthma):TI AND (attack):TI
30	(asthma):TI AND (acute):TI AND (worsen):TI
31	(COPD):TI AND (acute):TI AND (worsen):TI
32	(chronic obstructive):TI AND (exacerbation):TI
33	(pulmonary):TI AND (exacerbation):TI AND (disease):TI
34	(upper respiratory tract infection):TI OR (upper respiratory infection):TI OR (urti OR uri):TI
35	(lower respiratory tract infection):TI OR (lower respiratory infection):TI OR (lrti OR lri):TI
36	(respiratory tract infection):TI OR (respiratory infection):TI OR (rti OR ri):TI
37	(acute respiratory tract infection):TI OR (acute respiratory infection):TI OR (arti OR ari):TI
38	#24 OR #25 OR #26 OR #27 OR #28 OR #29 OR #30 OR #31 OR #32 OR #33 OR #34 OR #35 OR #36 OR #37
39	#5 OR #6 OR #7 OR #8 OR #9 OR #10 OR #11 OR #12 OR #13 OR #14 OR #15 OR #16 OR #17 OR #18 OR #19 OR #20 OR #21 OR #22 OR #23
40	#1 OR #2
41	#3 OR #4
42	#40 NOT #41
**43**	**#38 AND #39 AND #42**

Table Inclusion and Exclusion Criteria

S2

Inclusion Criteria

- Papers reporting empirical data related to cost of ARI management, i.e., Cost of Illness studies
- All disease spectra of ARI including RSV and influenza infections
- Period of publication 2000-2021
- Older adults aged ≥50 years
- Control arm of vaccine trials and cost-effectiveness analysis with empirical cost data
- Populations with or without known comorbidities

Exclusion Criteria

- Review articles
- Conference abstracts
- Articles prior to year 2000
- Vaccine trials and cost-effectiveness analysis not containing a control arm with empirical cost data
- Model-based economic evaluation without empirical data on costs related to ARI management
- Studies reporting costs only related to hospital-acquired pneumonia

Table Quality Assessment Criteria

1. The question and perspective are clearly stated
2. It is a cost study
3. Quantity of resources used and unit costs are reported separately
4. Data sources are clearly reported
5. Currency and price (including price year) are documented

6. Time horizon of costs is stated
7. Consideration of discounting is documented and justified
8. Details of statistical tests and result errors are clearly described
9. Sensitivity analysis is carried out (and adjustments stated)
10. A comparison is made between two alternatives
11. A standard definition or diagnosis of ARI is used
12. The answer to the study question is clearly stated (and valid)
13. Conclusions are drawn and relevant limitations are raised

Table Risk of bias checklist

No.	Criteria	Yes	No
1	Is the study population clearly described?		
2	Are competing alternatives clearly described?		
3	Is a well-defined research question posed in answerable form?		
4	Is the economic study design appropriate to the stated objective?		
5	Is the chosen time horizon appropriate in order to include relevant costs and consequences?		
6	Is the actual perspective chosen appropriate?		
7	Are all important and relevant costs for each alternative identified?		
8	Are all costs measured appropriately in physical unites?		
9	Are costs valued appropriately		
10	Are all important and relevant outcomes for each alternative identified?		

11	Are all outcomes measured appropriately?		
12	Are outcomes valued appropriately?		
13	Is an incremental analysis of costs and outcomes of alternatives performed?		
14	Are all future costs and outcomes discounted appropriately?		
15	Are all important variables, whose values are uncertain, appropriately subjected to sensitivity analysis?		
16	Do the conclusions follow from the data reported?		
17	Does the study discuss the generalizability of the results to other settings and patient/client groups?		
18	Does the article indicate that there is no potential conflict of interest of study researcher(s) and funders(s)?		

19	Are ethical and distributional issues discussed appropriately?		

Studies were considered high-quality if more than 15 points were addressed (≥75% fulfilment), medium-quality studies covered 10–14 points (50–74% fulfilment) and low-quality studies addressed a maximum of 9 points (<50%).

Table S5 Descriptive summary of eligible ARI-related cost management studies

Author<br>Year	Country	Type of EE	Study Design & Data source	Perspective	Condition	N	Male (%)	Age mean<br>(SD) /<br>% total	Reported comorbidities
**Baldo 2014 [1]**	Italy	COI	Retrospective observational cohort<br>Surveillance	Healthcare	Pneumonia<br>(CAP)	110,927	54%	≥65 yrs: 68.9%	Heart diseases: 18.1–27.8%<br>COPD and asthma: 11.5–15.0%<br>Diabetes mellitus: 11.3–15.4%<br>Dementia: 6.7–15.4%<br>Stroke: 7.0–11.0%<br>Renal disease: 7.0–8.8%<br>Cancer: 9.1–4.8%
**Bartolome 2004[2]**	Spain	COI	Prospective Cohort<br>EHR	Healthcare<br>(provider)	Pneumonia<br>(CAP)	224	Inpatients: 63%<br>Outpatients: 55%	>65 years<br>Inpatients: 50%<br>Outpatients: 9%	Chronic bronchitis: 65%
**Monge 2001[3]**	Spain	COI	Retrospective observational cohort<br>EHR (medical records)	Healthcare	Pneumonia<br>(CAP)	51,415	NS	NS	NS
**Costa 2017[4]**	France	COI	Prospective observational cohort<br>EHR	Healthcare<br>(Payer)	Pneumonia<br>(NHAP)	345	20%	87.8 (6.3) yrs	CVD: 66%<br>DM9 14%

Author<br>Year	Country	Type of EE	Study Design & Data source	Perspective	Condition	N	Male (%)	Age mean<br>(SD) /<br>% total	Reported comorbidities
			(claims records)						Other metabolic diseases 31%<br>Depression: 36%<br>Pain: 38%<br>Alzheimer disease: 30%<br>Other dementia: 30%
**Personne 2016[5]**	France	COI	Prospective observational cohort<br>EHR<br>(claims records)	Healthcare	Pneumonia<br>(CAP)	886	NS	NS	NS
**Dupuis 2020[6]**	France	COI	Retrospective observational	Health care	Pneumonia<br>(CAP)	1,271	67%	65(NS)<br>/76.3%	CRD:51.2%<br>DM:24.9%<br>CHF:31/3%<br>Renal disease: 14.4%<br>Liver disease: 16.3%
**Rozenbaum[7]**	Netherlands	COI	Prospective observational cohort<br>EHR<br>(claims records)	Healthcare<br>(Payer)	Pneumonia<br>(CAP)	195,372	NS	NS	NS

Author Year	Country	Type of EE	Study Design & Data source	Perspective	Condition	N	Male (%)	Age mean (SD) / % total	Reported comorbidities
**Vissink 2016[8]**	Netherlands	COI	Prospective Cohort CRF	Healthcare	Pneumonia (CAP)	0	NS	NS	NS
**Tichopad 2013[9]**	Czech Republic, Slovakia, Poland, Hungary	COI	Retrospective observational cohort (EHR medical records, claims)	Societal	Pneumonia (CAP) ICD-9	291883	NA	NA	NA
**Carriere 2004[10]**	Canada	COI	Retrospective observational cohort EHR (claims records)	Healthcare (Payer / Province)	Pneumonia (CAP)	43,642	NS	65 (NS) yrs	NS
**Ye 2008[11]**	USA	CEA	Retrospective observational cohort EHR (medical records)	Healthcare (Payer)	Pneumonia (CAP) ICD-9	7526	49.4-52.5%	≥50 yrs: 59.06(7.45) /39.4% ≥65 yrs: 71.92(6.51) /5.7%	Respiratory comorbidities: 48-55% Cardiovascular comorbidities: 18-36% Diabetes: 14.2-19.1%

**Broulette**<br>**2013[12]**	USA	COI	Retrospective observational cohort<br>(Insurance database)	Societal<br>(Third party payer)	Pneumonia<br>(CAP) ICD-9, pneumonia and chest X-ray claim	59535	45.6%	≥50 yrs: 48%	Diabetes: 13.2%<br>Congestive heart failure: 3.8%<br>Coronary artery disease: 6.1%<br>COPD: 7.7%<br>Asthma: 8.3%
**Kaplan**	USA	COI	Retrospective observational cohort	Healthcare	Pneumonia	623718	46.4%	77 yrs	Congestive heart failure: 31.9%

**Author**<br>**Year**	**Country**	**Type of EE**	**Study Design & Data source**	**Perspective**	**Condition**	**N**	**Male (%)**	**Age mean (SD) / % total**	**Reported comorbidities**
**2002[13]**			EHR (medical records)		(CAP) ICD-9				Pulmonary disease: 24.5%<br>Diabetes mellitus: 17.4%<br>Malignancy: 9.1%<br>Neurologic disease: 8.6%<br>Myocardial infarction: 5.4%<br>Renal disease: 2.7%<br>Liver disease: 0.6%

**Olasupo 2018[14]**	USA	COI	Retrospective cross-sectional study (EHR medical records)	Healthcare	Pneumonia (CAP) ICD-9	269961	48%	79.7(8.0) yrs	Congestive heart failure: 26.4%<br>Chronic pulmonary disease: 49.1%<br>Hypertension: 66.9%<br>Liver disease: 1.5%<br>Renal failure: 18.6%<br>Metastatic cancer: 3.9%
**Brown 2018[15]**	USA	COI	Retrospective cohort (Insurance database)	Healthcare (Payer) Patient	Pneumonia (CAP) ICD-9	15701	42.2%	75.9 yrs	Diabetes: 25.0%<br>Chronic pulmonary disease: 14.7%<br>Coronary artery disease: 14.2%<br>Renal disease: 11.3%
**Curns 2008[16]**	USA	COI	Retrospective observational cohort	Healthcare	ARI	12,400,000	NS	NS	NS

**Author Year**	**Country**	**Type of EE**	**Study Design & Data source**	**Perspective**	**Condition**	**N**	**Male (%)**	**Age mean (SD) / % total**	**Reported comorbidities**
			EHR (medical records)						

**Kruse 2003[17]**	USA	CMA	Prospective cohort<br>Medical Records	Societal	Pneumonia<br>(NHAP)	502	31%	60–69 yrs: 3.4%<br>70–79 yrs: 16.3%<br>80–89 yrs: 44.8%<br>90 yrs: 35.5%	CHF: 33.3%<br>COPD: 20.9%<br>CVA: 29.3%<br>Dementia: 61.6%<br>Depression: 39.4%<br>DM: 18.5%:<br>Pressure ulcers 14.7%
**Sato 2013[18]**	USA	COI	Prospective observational cohort<br>EHR<br>(claims records)	Healthcare<br>(Payer)	Pneumonia<br>(CAP)	27,659	50%	62.6 (10.1) yrs	CHF: 9.91%<br>DM:17.9%<br>Asthma: 10.5%<br>COPD: 21.1%<br>CAD: 14.8%
**Park 2015[19]**	USA	COI	Retrospective observational cohort<br>Survey Data	Healthcare<br>(Payer)	Pneumonia	1,790	49%	<5 yrs: 1.4 (0.1) yrs<br>5 -<18yrs: 9.3 (0.3) yrs<br>≥18-<45yrs: 33.2 (0.5) yrs	NS

**Author Year**	**Country**	**Type of EE**	**Study Design & Data source**	**Perspective**	**Condition**	**N**	**Male (%)**	**Age mean (SD) / % total**	**Reported comorbidities**

								≥45 <65yrs: 54.7 (0.4) yrs<br>≥65yrs: 75.4 (0.5) yrs	
**Thomas 2012[20]**	USA	COI	Prospective observational cohort<br>EHR<br>(claims records)	Healthcare<br>(Payer)	Pneumonia (CAP & HAP)	1,908,928	37%	65-74 yrs: 47.5%<br>75-84 yrs: 37.6%<br>85 yrs: 14.9%	COPD: 14.2%<br>CHF 14.3 %<br>DM: 23.8%<br>Dementia / Senility: 10.4%<br>Asthma: 5.0%
**Weycker 2016[21]**	USA	COI	Retrospective observational cohort<br>EHR<br>(claims records)	Healthcare	Pneumonia	1,933	NS	NS	CHD: 52 %<br>DM: 50%<br>Chronic Lung Disease: 19 %
**Yu 2012[22]**	USA	COI	Retrospective observational cohort<br>EHR<br>(claims records)	Healthcare	Pneumonia<br>(CAP)	62,440	44%	75.0 (NS) yrs	CAD: 34.9%<br>CHF: 35.1%<br>DM: 34.1%<br>Asthma: 5.0%<br>COPD: 46.9%
**Hui[23]**	Hong Kong	COI	Retrospective observational cohort<br>EHR	Healthcare	ILI	259	NS	NS	NS

Author<br>Year	Country	Type of EE	Study Design & Data source	Perspective	Condition	N	Male (%)	Age mean<br>(SD) /<br>% total	Reported comorbidities
			(medical records)						
**Konomura 2017[24]**	Japan	COI	Retrospective cohort (HER, administrative database)	Healthcare	Pneumonia (CAP) ICD-10	29619	61%	80.1(8.4)yrs	DM: 11%<br>COPD: 12%<br>Dementia: 1%<br>Dialysis: 2%<br>Liver function failure: 15%<br>Rheumatism: 3%<br>Cancer: 9%
**Song 2013[25]**	South Korea	COI	Retrospective observational cohort<br>EHR<br>(linked medical / claims records)	Healthcare	Pneumonia<br>(invasive pneumococcal disease)	970	NS	18–64 yrs: 55.2%<br>65–74 yrs: 27.4%<br>≥75 yrs: 17.4%	COPD: 7.2–16.6%
**Yoo 2013[26]**	South Korea	COI	Prospective observational cohort<br>EHR<br>(claims records)	Healthcare<br>(Payer)	Pneumonia	693	57%	70.1 (10.5) yrs	NS

**Akyil 2015[27]**	Turkey	COI	Prospective observational cohort<br>EHR<br>(claims records)	Healthcare	Pneumonia<br>(CAP)	87	76%	69 (14) yrs	COPD: 64%<br>Malignancy: 16%<br>DM: 16%

**Author**<br>**Year**	**Country**	**Type of EE**	**Study Design & Data source**	**Perspective**	**Condition**	**N**	**Male (%)**	**Age mean (SD) / % total**	**Reported comorbidities**
									CHF: 16%
**Kosar 2017[28]**	Turkey	COI	Retrospective observational cohort<br>EHR (medical records)	Healthcare	Pneumonia (CAP) ICD-10	162	53%	≥65 yrs: 38.6%<br>Inpatients: 48.6%<br>Outpatients:28.9%	Heart diseases: 2.8–12.0%<br>COPD: 22.7–36.5%<br>Asthma: 6.7-20.4%<br>Diabetes mellitus: 11.3–15.4%<br>Hypertension: 7.1-18.3%
**Peasah 2015[29]**	India	COI	Prospective cohort<br>Survey	Societal	ARI	259	43%	≤5 yrs: 34%<br>6-17 yrs: 27%<br>18-64 yrs: 31%<br>≥65 yrs: 8%	NS

**Chen 2020[30]**	Japan	COI	Retrospective observational cohort<br>EHR (medical records)	Health care	Pneumonia	325	65.20%	80(MEDIAN<br>RANGE74-85)	Dementia 12.6% bedsores 1.85% aspiration 11.7%
**Vo 2018[31]**	Vietnam	COI	Prospective, Incidence-based	Societal	Pneumonia	255	58%	66 y (16)<br>45-64 y (29.4%)<br>65-74 y (23.1%)<br>75-84y (30.6%)<br>≥85y (8.3%)	Cardiovascular comorbidities: 69%<br>digestive diseases 45.1%<br>Pulmonary disease: 41.4%

**Author Year**	**Country**	**Type of EE**	**Study Design & Data source**	**Perspective**	**Condition**	**N**	**Male (%)**	**Age mean (SD) / % total**	**Reported comorbidities**
								≥65 y (62%)<br>≥75y (38.9%)	

Author Year	Country	Type of EE	Study Design & Data source	Perspective	Condition	N	Male (%)	Age mean (SD) / % total	Reported comorbidities
**Gumus 2019[32]**	Turkey	COI	retrospective cohort study	Healthcare	CAP	291	38.80%	68.4±16.8<br><50y 13.4%<br>50-59 y (7.2%)<br>60-69y (25%)<br>70-79y (25%)<br>≥80y (29.2%)	COPD 45.7% DM 23%<br>Afib 14.3%<br>Hypertension 42.3% chronic kidney insufficiency 8.2% chronic liver 2.4% cerebrovascular event 12% hyperlipidemia 4.5% coronary artery disease 15.5% arrhythmia 7.6% CHF 25.1% valve disease3.1%
**Han 2021[33]**	China	CEA	retrospective, matched study	Healthcare	CAP	511	43.80%	≥65y (100%)	underlying disease 91.9% chronic respiratory disease 28.9% chronic cardiac disease 63% chronic liver 1.6%

									chronic renal disease 5.2%<br>Diabetes Mellitus 15.9%<br>cerebrovascular disease 27% risk<br>of aspiration 9.2%<br>Long-term bedridden 9.4%
**Sakamoto 2021[34]**	Japan	COI	Retrospective matched-pair cohort study national inpatient database retrospective matched cohort design	healthcare	Compared non-MRSA CAP pneumonia to MRSA pneumonia	3102 MRSA<br>12,320 non-MRSA	66.40%	79.4 y (10.8)	NS
**Weycker 2020[35]**	USA	COI	retrospective matched cohort design<br>US private healthcare claims repository	Healthcare (Payer/Provider)	Pneumonia<br>(CAP)	43975	47%	≥65y (56%)<br>≥75y (39%)<br>18-49y (15.5%)<br>50-64y (28.9%)<br>65-74y (16.3%)	"AT RISK" 40%<br>Chronic cardiac disease 25%<br>COPD 15%<br>DM 27%<br>Chronic lung disease 5.2% liver disease 0.9%
**Meier 2020[36]**	UK	COI	retrospective observational database study	cost perspective	Influenza, ARI or	156,193	NS	≥65y 15%	NS

Author<br>Year	Country	Type of EE	Study Design & Data source	Perspective	Condition	N	Male (%)	Age mean<br>(SD) /<br>% total	Reported comorbidities
			Clinical Practice Research Datalink<br>(CPRD)/Health Episode Statistics (HES)<br>dataset	was that of the NHS	Influenza-related respiratory infection				
**Moss 2020[37]**	England	COI	Retrospective cohort study Hospital Episode Statistics (HES) database	healthcare<br>(NHS)	Influenza	2017/18 flu season<br>17565 <65<br>7320 (65-74y)<br>16060 (>75y)<br>2018/19 flu season<br>21570 (<65)<br>4975 (65-74y)<br>8495 (>75y)	NS	NS	NS
**Prasad 2020[38]**	New Zealand	COI	Retrospective cohort study	healthcare	ARI (RSV)	8776	46.60%	18-49Y 2117<br>50-64 Y 1974<br>65-79Y 2677<br>>65 4685<br>≥80 2008	NS

**Scholz 2019[39]**	Germany	COI	Claims data from a large German statutory sickness fund	healthcare	influenza	95089	49%	35-59y 39,531<br>>60 9217	NS

**Author**<br>**Year**	**Country**	**Type of EE**	**Study Design & Data source**	**Perspective**	**Condition**	**N**	**Male (%)**	**Age mean (SD) / % total**	**Reported comorbidities**
**Yoon 2020[40]**	S. Korea	COI	Retrospective cohort study	healthcare	RSV	204	46.30%	50-64yrs 52<br>>65yrs 132	any 89.7%<br>Diabetes 30.2%<br>Cardiovascular disease 26.8%<br>Stroke 20.4%<br>Respiratory disease 27.8%<br>Chronic kidney disease 15.6%<br>Liver disease 7.3%<br>Solid cancer 22.4%<br>Hematologic malignancy 5.8%

Author Year	Country	Type of EE	Study Design & Data source	Perspective	Condition	N	Male (%)	Age mean (SD) / % total	Reported comorbidities
**Choi 2021[41]**	USA	COI	Prospective surveillance for RSV infection	Healthcare	RSV	601	41%	50-64Y (29%)<br>>65Y (57%)	Chronic lung disease 49%<br>cardiovascular 56%<br>diabetes mellitus 43%<br>chronic kidney disease 28%<br>chronic liver disease 7%<br>obesity 30%
**Bradley 2020[42]**	USA	COI	Prospective surveillance for RSV infection	Healthcare	RSV or influenza	2090	41.5%-49.6%	77.7-78.4 yrs	Chronic lung disease:26.2%-38.7%<br>Congestive heart failure 27.6%-40. 6%56%<br>diabetes mellitus 39.4%-49.1%
**Author Year**	**Country**	**Type of EE**	**Study Design & Data source**	**Perspective**	**Condition**	**N**	**Male (%)**	**Age mean (SD) / % total**	**Reported comorbidities**
									Cancer 8.4%-11.2%

COI- Cost Of Illness; EE: Economic Evaluation ; CAP: Community acquired pneumonia

Table S6 Quality, Bias and Transferability Summary of Included Studies

Study Characteristics	Categories	Mean N (Percentage of Total) or Mean Percentage ± Standard Deviation
Quality Assessment Score	Overall	76.98% ± 14.07%
	European Region	73.48% ± 15.63%
	The Americas Region	82.50% ± 11.92%
	West Pacific Region	74.44% ± 13.80%
	South East Asia Region	66.67% ± 0.00%
Potential for Bias	High Quality (Low Potential for Bias)	21 (50.00%)
	Medium Quality (Medium Potential for Bias)	20 (47.62%)
	Low Quality (High Potential for Bias)	1 (2.38%)

Table S7 Quality and Potential for Bias Results

Author	Quality<br>Drummond score, %		Potential for Bias
Bradley 2020[42]	7	77.78%	Medium
Akyil 2015[27]	5	55.56%	Medium
Baldo 2014[1]	5	55.56%	Medium
Bartolome 2004[2]	8	88.89%	Low
Broulette 2013[12]	9	90.00%	Low
Brown 2018[15]	5	55.56%	Low
Carriere 2004[10]	7	77.78%	Low
Chen 2020[30]	5	50.00%	Medium
Choi 2021[41]	9	100.00%	Low
Costa 2017[4]	8	88.89%	Low
Curns 2008[16]	7	77.78%	Medium
Vo 2018[31]	8	88.89%	Low
Dupuis 2021[6]	7	77.78%	Low
Gumus 2019[32]	5	55.56%	Medium
Han 2021[33]	7	70.00%	Low
Hui 2008[23]	6	66.67%	Medium
Kaplan 2002[13]	6	66.67%	Medium
Konomura 2017[24]	8	88.89%	Low
Kosar 2017a[28]	8	80.00%	Low
Kruse 2003[17]	8	88.89%	Low
Meier 2020[36]	8	88.89%	Low

Monge 2001[3]	5	55.56%	Medium
Moss 2020[37]	7	77.78%	Medium
Olasupo 2018[14]	7	77.78%	Medium
Park 2015[19]	8	88.89%	Low
Peasah 2015[29]	6	66.67%	Medium
Personne 2016[5]	7	77.78%	Low
Prasad 2020[38]	8	88.89%	Low
Rozenbaum 2015[7]	7	77.78%	Medium
Sakamoto 2021[34]	8	80.00%	Low
Sato 2013[18]	8	88.89%	Medium
Scholz 2019[39]	8	88.89%	Medium
Song 2013[25]	7	77.78%	Medium
Thomas 2012[20]	9	90.00%	Medium
Tichopad 2013[9]	4	44.44%	High
Vissink 2016[8]	8	88.89%	Medium
Weycker 2016[21]	9	100.00%	Medium
Weycker 2021[35]	8	80.00%	Low
Ye 2008[11]	7	70.00%	Medium
Yoo 2013[26]	5	55.56%	Low
Yoon 2020[40]	7	77.78%	Low
Yu 2012[22]	9	90.00%	Low

Supplementary Material References

1 Baldo V, Cocchio S, Baldovin T, Buja A, Furlan P, Bertoncello C, et al. A population-based study on the impact of hospitalization for pneumonia in different age groups. BMC infectious diseases. 2014;14:485.

2 Bartolome M, Almirall J, Morera J, Pera G, Ortun V, Bassa J, et al. A population-based study of the costs of care for community-acquired pneumonia. European Respiratory Journal. 2004;23:610-6.

3 Monge V, San-Martin VM, Gonzalez A. The burden of community-acquired pneumonia in Spain. European journal of public health. 2001;11:362-4.

4 Costa N, Hoogendijk EO, Mounie M, Bourrel R, Rolland Y, Vellas B, et al. Additional

Cost Because of Pneumonia in Nursing Home Residents: Results From the Incidence of Pneumonia and Related Consequences in Nursing Home Resident Study. Journal of the American Medical Directors Association. 2017;18:453.e7-.e12.

5 Personne V, Chevalier J, Buffel du Vaure C, Partouche H, Gilberg S, de Pouvourville G. CAPECO: Cost evaluation of community acquired pneumonia managed in primary care. Vaccine. 2016;34:2275-80.

6 Dupuis C, Sabra A, Patrier J, Chaize G, Saighi A, Feger Cl, et al. Burden of pneumococcal pneumonia requiring ICU admission in France: 1-year prognosis, resources use, and costs. Critical Care. 2021;25.

7 Rozenbaum MH, Mangen MJ, Huijts SM, van der Werf TS, Postma MJ. Incidence, direct costs and duration of hospitalization of patients hospitalized with community acquired pneumonia: A nationwide retrospective claims database analysis. Vaccine. 2015;33:3193-9.

8 Vissink CE, Huijts SM, de Wit GA, Bonten MJ, Mangen MJ. Hospitalization costs for community-acquired pneumonia in Dutch elderly: an observational study. BMC infectious diseases. 2016;16:466.

9 Tichopad A, Roberts C, Gembula I, Hajek P, Skoczynska A, Hryniewicz W, et al. Clinical and economic burden of community-acquired pneumonia among adults in the Czech Republic, Hungary, Poland and Slovakia. PloS one. 2013;8:e71375.

10 Carriere KC, Jin Y, Marrie TJ, Predy G, Johnson DH. Outcomes and costs among seniors requiring hospitalization for community-acquired pneumonia in Alberta. Journal of the American Geriatrics Society. 2004;52:31-8.

11 Ye X, Sikirica V, Schein JR, Grant R, Zarotsky V, Doshi D, et al. Treatment failure rates and health care utilization and costs among patients with community-acquired pneumonia treated with levofloxacin or macrolides in an outpatient setting: a retrospective claims database analysis. Clinical therapeutics. 2008;30:358-71.

12 Broulette J, Yu H, Pyenson B, Iwasaki K, Sato R. The incidence rate and economic burden of community-acquired pneumonia in a working-age population. American health & drug benefits. 2013;6:494-503.

13 Kaplan V, Angus DC, Griffin MF, Clermont G, Scott Watson R, Linde-Zwirble WT. Hospitalized community-acquired pneumonia in the elderly: age- and sex-related patterns of care and outcome in the United States. American journal of respiratory and critical care medicine. 2002;165:766-72.

14 Olasupo O, Xiao H, Brown JD. Relative Clinical and Cost Burden of Community-Acquired Pneumonia Hospitalizations in Older Adults in the United States-A Cross-Sectional Analysis. Vaccines. 2018;6.

15 Brown JD, Harnett J, Chambers R, Sato R. The relative burden of community-acquired pneumonia hospitalizations in older adults: a retrospective observational study in the United States. BMC geriatrics. 2018;18:92.

16 Curns AT, Steiner CA, Sejvar JJ, Schonberger LB. Hospital charges attributable to a primary diagnosis of infectious diseases in older adults in the United States, 1998 to 2004. Journal of the American Geriatrics Society. 2008;56:969-75.

17 Kruse RL, Boles KE, Mehr DR, Spalding D, Lave JR. The cost of treating pneumonia in the nursing home setting. Journal of the American Medical Directors Association. 2003;4:81-9.

18 Sato R, Gomez Rey G, Nelson S, Pinsky B. Community-acquired pneumonia episode costs by age and risk in commercially insured US adults aged >/=50 years. Applied health economics and health policy. 2013;11:251-8.

19 Park H, Adeyemi AO, Rascati KL. Direct Medical Costs and Utilization of Health Care Services to Treat Pneumonia in the United States: An Analysis of the 2007-2011 Medical Expenditure Panel Survey. Clinical therapeutics.

2015;37:1466-76.e1.
20 Thomas CP, Ryan M, Chapman JD, Stason WB, Tompkins CP, Suaya JA, et al. Incidence and cost of pneumonia in medicare beneficiaries. Chest. 2012;142:973-81.
21 Weycker D, Farkouh RA, Strutton DR, Edelsberg J, Shea KM, Pelton SI. Rates and costs of invasive pneumococcal disease and pneumonia in persons with underlying medical conditions. BMC health services research. 2016;16:182.
22 Yu H, Rubin J, Dunning S, Li S, Sato R. Clinical and economic burden of community-acquired pneumonia in the Medicare fee-for-service population. Journal of the American Geriatrics Society. 2012;60:2137-43.
23 Hui DS, Woo J, Hui E, Foo A, Ip M, To KW, et al. Influenza-like illness in residential care homes: a study of the incidence, aetiological agents, natural history and health resource utilisation. Thorax. 2008;63:690-7.
24 Konomura K, Nagai H, Akazawa M. Economic burden of community-acquired pneumonia among elderly patients: a Japanese perspective. Pneumonia (Nathan Qld). 2017;9:19.
25 Song JY, Choi JY, Lee JS, Bae IG, Kim YK, Sohn JW, et al. Clinical and economic burden of invasive pneumococcal disease in adults: a multicenter hospital-based study. BMC infectious diseases. 2013;13:202.
26 Yoo KH, Yoo CG, Kim SK, Jung JY, Lee MG, Uh ST, et al. Economic burden and epidemiology of pneumonia in Korean adults aged over 50 years. Journal of Korean medical science. 2013;28:888-95.
27 Akyıl FT, Hazar A, Erdem İ, Öneş CP, Yalçınsoy M, Irmak İ, et al. Hospital Treatment Costs and Factors Affecting These Costs in Community-Acquired Pneumonia. Turkish Thoracic Journal. 2015;16.
28 Kosar F, Alici DE, Hacibedel B, Arpinar Yigitbas B, Golabi P, Cuhadaroglu C. Burden of community-acquired pneumonia in adults over 18 y of age. Human vaccines & immunotherapeutics. 2017;13:1673-80.
29 Peasah SK, Purakayastha DR, Koul PA, Dawood FS, Saha S, Amarchand R, et al. The cost of acute respiratory infections in Northern India: a multi-site study. BMC public health. 2015;15:330.

30 Chen H, Hara Y, Horita N, Saigusa Y, Hirai Y, Kaneko T. Declined Functional Status Prolonged Hospital Stay for Community-Acquired Pneumonia in Seniors. Clinical Interventions in Aging. 2020;15:1513 - 9.

31 Vo DTQ, editor Socioeconomic Burden of Community-acquired Pneumonia Associated Hospitalizations among Vietnamese Patients: A Prospective, Incidence-based Study2018.

32 Gümüş A, Çilli A, Çakın Ö, Karakurt Z, Ergan B, Aksoy E, et al. Factors Affecting Cost of Patients with Severe Community-Acquired Pneumonia in Intensive Care Unit. Turkish thoracic journal. 2019:1-8.

33 Han X, Chen L, Wang Y, Li H, Wang H, Xing X, et al. Cost Effectiveness of Different Initial Antimicrobial Regimens for Elderly Community-Acquired Pneumonia Patients in General Ward. Infection and Drug Resistance. 2021;14:1845 - 53.

34 Sakamoto Y, Yamauchi Y, Jo T, Michihata N, Hasegawa WT, Takeshima H, et al. In-hospital mortality associated with community-acquired pneumonia due to methicillin-resistant Staphylococcus aureus: a matched-pair cohort study. BMC Pulmonary Medicine. 2021;21.

35 Weycker D, Moynahan A, Silvia A, Sato R. Attributable Cost of Adult Hospitalized Pneumonia Beyond the Acute Phase. PharmacoEconomics Open. 2020;5:275 - 84.

36 Meier GC, Watkins J, Mcewan P, Pockett RD. Resource use and direct medical costs of acute respiratory illness in the UK based on linked primary and secondary care records from 2001 to 2009. PloS one. 2020;15:e0236472.

37 Moss JWE, Davidson C, Mattock R, Gibbons I, Mealing S, Carroll SM. Quantifying the direct secondary health care cost of seasonal influenza in England. BMC public health. 2020;20.

38 Prasad N, Newbern EC, Trenholme AA, Thompson MG, Grant CC. The health and economic burden of respiratory syncytial virus associated hospitalizations in adults. PloS one.15:e0234235.

39 Scholz S, Damm O, Schneider U, Ultsch B, Wichmann O, Greiner W. Epidemiology and cost of seasonal influenza in Germany - a claims data analysis. BioMed Central. 2019.

40 Yoon JG, Noh JY, Choi WS, Park JJ, Kim WJ. Clinical characteristics and disease burden of respiratory syncytial virus infection among hospitalized adults.

Scientific Reports. 2020;10:12106.
41 Choi Y, Hill-Ricciuti AC, Branche AR, Sieling WD, Saiman L, Walsh EE, et al. Cost determinants among adults hospitalized with respiratory syncytial virus in the United States, 2017–2019. Influenza and Other Respiratory Viruses. 2021;16:151 - 8.
42 Bradley A, Jaejin A, Sy LS, Zendi S, Jeff S, Hung-Fu T. Cost of Hospitalization Associated With Respiratory Syncytial Virus Infection Versus Influenza Infection in Hospitalized Older Adults. The Journal of infectious diseases. 2020;222:6.
